# Survival of radioiodine treated hyperthyroid cats that are euthyroid and hypothyroid after treatment, and effect of levothyroxine supplementation on survival time of cats with iatrogenic hypothyroidism

**DOI:** 10.1111/jvim.17295

**Published:** 2025-01-20

**Authors:** Sarah E. Cox, Jennifer Wakeling, Teresa Hall, Tim L. Williams

**Affiliations:** ^1^ Department of Veterinary Medicine University of Cambridge Cambridge UK; ^2^ Douglas College Coquitlam British Columbia Canada; ^3^ North West Nuclear Medicine Vancouver British Columbia Canada

**Keywords:** azotemia, blood pressure, chronic kidney disease, feline

## Abstract

**Background:**

Hyperthyroid cats that are azotemic and hypothyroid after surgical or medical treatment have poor outcomes, and supplementation with levothyroxine (LT4) improves survival. However, the effect of LT4 supplementation on survival of nonazotemic, hypothyroid radioiodine (RI)‐treated hyperthyroid cats is unknown.

**Hypothesis:**

Radioiodine treated hyperthyroid cats with iatrogenic hypothyroidism or azotemia have shorter survival times than euthyroid, nonazotemic cats and supplementation of LT4 improves survival times of hypothyroid cats.

**Animals:**

One hundred seventeen RI treated hyperthyroid cats.

**Methods:**

Prospective cohort study. Radioiodine treated cats were screened for azotemia and iatrogenic hypothyroidism using TSH stimulation test; LT4 supplementation was offered to all hypothyroid cats with decision to treat based on owner preference. The log rank test was used to compare survival times between groups, and the Mann‐Whitney *U* test was used to compare age and renal variables. Data are presented as median [range].

**Results:**

Euthyroid azotemic cats (934 [759‐2035] days) and nonsupplemented hypothyroid cats (azotemic and nonazotemic combined, 1232 [238‐2363] days) had shorter survival times than euthyroid nonazotemic cats (1616 [663‐3369] days, *P* = .003 and *P* = .002, respectively). Levothyroxine supplemented hypothyroid nonazotemic cats had longer survival times than nonsupplemented hypothyroid nonazotemic cats (1037 [300‐2401] days vs 768 [34‐1014] days; *P* = .027). Levothyroxine supplementation was not associated with prolonged survival times in hypothyroid azotemic cats vs nonsupplemented hypothyroid azotemic cats (771 [718‐1558] days vs 152 [82‐1852] days, respectively, *P* = .991).

**Conclusions and Clinical Importance:**

Levothyroxine supplementation in nonazotemic cats with iatrogenic hypothyroidism (diagnosed based on TSH stimulation test results) improved survival times, although randomized controlled trials are needed.

**Abbreviations**
CKD: chronic kidney disease
DVM: Doctor of Veterinary Medicine
fT4: free‐thyroxine
GFR: glomerular filtration rate
HR: hazard ratio
LT4: levothyroxine
RI: radioiodine I131
SBP: systolic blood pressure
T3: total tri‐iodothyronine
TSH: thyroid stimulating hormone
tT4: total‐thyroxine
UPC: urine protein : creatinine ratio
USG: urine‐specific gravity

## 1 INTRODUCTION

Hyperthyroidism is the most commonly diagnosed geriatric endocrine disease, estimated to develop in over 10% of senior cats.^1^ Radioiodine (RI) treatment is often cited as the preferred treatment option,^2^ and median survival times of cats treated with RI range between 2 and 4 years.^3^, ^4^, ^5^, ^6^, ^7^ Treatment of hyperthyroidism can unmask azotemia associated with chronic kidney disease (CKD) because of the decline in glomerular filtration rate (GFR) after normalization of thyroid hormone concentrations,^8^ although the development of azotemia after treatment (with antithyroid medication or by thyroidectomy) is not associated with a reduced survival time in cats that remain euthyroid after treatment.^9^ However, 1 study has reported that post treatment serum creatinine concentrations in RI treated cats are associated with shorter survival times after adjustment for age, which could suggest that concurrent azotemia does affect survival of cats that are euthyroid after RI treatment.^6^

Iatrogenic hypothyroidism can also further reduce GFR,^8^ and cats with iatrogenic hypothyroidism have an increased risk of azotemia compared to their euthyroid counterparts.^9^ Furthermore, hypothyroid azotemic cats have shorter survival times compared to hypothyroid nonazotemic cats which suggests that the combination of iatrogenic hypothyroidism and azotemia is detrimental.^9^ This could reflect progression of renal disease because iatrogenic hypothyroidism is associated with a lower PCV^9^ and increased systolic blood pressure (SBP).^10^ Decreased PCV is a known risk factor for CKD progression^11^ whilst raised SBP is documented to increase the risk of proteinuria which, in turn, is independently associated with the development of azotemia and shorter survival times in cats with CKD.^12^, ^13^, ^14^ Proteinuria is also associated with overall survival time in cats with hyperthyroidism^15^ although the strength of this association is weaker than in cats with CKD.

Restoration of euthyroidism in hypothyroid azotemic cats will decrease plasma/serum creatinine concentrations and reduce the prevalence of concurrent azotemia.^16^, ^17^ However, only 1 study has shown an association between levothyroxine (LT4) supplementation and prolonged survival in azotemic hypothyroid cats,^17^ thus supporting a causal association between hypothyroidism and poorer outcomes. The effect of LT4 supplementation on survival times of nonazotemic cats with iatrogenic hypothyroidism has not been evaluated to date.

In the present study, we aimed to evaluate whether the presence of azotemia after RI treatment was associated with reduced survival time in cats defined as euthyroid based on the results of a thyroid stimulating hormone (TSH) stimulation test.^10^ Furthermore, we evaluated the survival times of cats defined as hypothyroid (based on TSH stimulation test) with and without azotemia. Finally, we report the effects of LT4 supplementation on survival of azotemic and nonazotemic RI treated cats with iatrogenic hypothyroidism.

## 2 MATERIALS AND METHODS

Hyperthyroid cats treated with RI at North West Nuclear Medicine (Vancouver, Canada) over a period of 4 years (2013‐2016) that underwent TSH stimulation testing as part of a previous study were eligible for inclusion.^10^ The exclusion criteria were: any animals suffering from an acute or severe illness, animals where sedation was required for examination, and animals that were on thyroid‐suppressive medication (minimum 6 week withdrawal) or an iodine restricted diet. Cats were enrolled at least 12 weeks after RI treatment and received a clinical examination and laboratory testing to evaluate renal function and thyroid status. Additional data collected for each cat included clinical history, physical examination findings, basic biochemistry and hematology testing, urinalysis, urine protein : creatinine ratio (UPC), TSH, free‐thyroxine (fT4), total‐thyroxine (tT4; before and after TSH stimulation), and measurement of systolic blood pressure (SBP) by Doppler sphygmomanometry.

### 2.1 Biochemical assays

Blood sampling was performed in the morning; blood was placed into serum separator tubes, allowed to clot for at least 20 minutes and then centrifuged within 1 hour. Samples were stored at 4°C to 6°C and analyzed within 24 hours.

Cats were classified by thyroid function according to results of TSH stimulation testing^10^; cats with post‐TSH stimulation tT4 <1.5 μg/dL (19 nmol/L) were classed as hypothyroid, cats with post‐TSH stimulation tT4 of 1.5 to 2.3 μg/dL (19‐30 nmol/L) and tT4 ratio <1.5 were classified as equivocal and cats with after‐stimulation tT4 ≥2.3 μg/dL (>30 nmol/L) or after‐stimulation tT4 1.5 to 2.3 μg/dL and before: after tT4 ratio ≥1.5 were classified as euthyroid. Serum tT4 concentrations were measured using a human tT4 enzyme immunoassay (Microgenics DRI) previously validated for use with feline serum^18^ and serum TSH concentrations were measured using a canine TSH assay (Siemens Immulite) that has also been validated for use with feline samples.^19^ Free T4 was measured using a semiautomated chemiluminescent immunoassay (CLIA) method for measuring fT4 with veterinary‐specific modification (IMMULITE 2000® Veterinary Free T4 (Catalogue no. L2KVF42), Siemens Healthcare Diagnostics). A second blood sample was taken for measurement of tT4 6 hours after IV administration of 0.05 mg rhTSH (THYROGEN thyrotropin alfa for injection, Genzyme Corp.).

Renal function was determined at the time of TSH stimulation testing by measurement of serum urea and creatinine concentrations and urine‐specific gravity (USG). Cats were classified as azotemic if their serum creatinine concentration was >2.5 mg/dL (upper limit of laboratory reference interval) with USG <1.035.

### 2.2 L‐thyroxine supplementation

LT4 supplementation was offered to all owners of hypothyroid cats, regardless of their azotemic status. Decision to treat was based on client preference and was not randomized. Initial LT4 dose was determined at the discretion of the attending veterinarian and was not standardized. Serum tT4 and TSH concentrations were reevaluated every 6 weeks, where possible, and LT4 dose was increased by the primary care DVM until the serum TSH concentration normalized, where possible.

### 2.3 Survival analysis

Baseline TSH stimulation testing was performed in 2015 to 2017. Cats were then followed‐up at yearly intervals until the date of death (all‐cause mortality) by contact with the primary care DVM or via email communication with the owner. The last round of follow‐up was performed in 2022. When a cat was lost to follow‐up the follow‐up period (days) was calculated from time of RI therapy up to the date they were last seen. Cats were censored from the survival analysis if they were alive at the end of the follow‐up period or if they were lost to follow‐up.

### 2.4 Statistical analysis

SPSS version 27 was used for statistical analysis. Survival times were calculated from the time of RI therapy to death (all‐cause mortality) using Kaplan‐Meier analysis. Survival times of cats were stratified at the time of TSH stimulation testing as euthyroid nonazotemic, euthyroid azotemic, equivocal nonazotemic, equivocal azotemic, hypothyroid supplemented (combined azotemic and nonazotemic) and hypothyroid nonsupplemented (combined azotemic and nonazotemic) and were compared using the Log rank test. Survival times of azotemic and nonazotemic hypothyroid cats that were and were not supplemented were also compared using the Log rank test. Multivariable Cox regression analysis was used to identify if azotemic status was independently associated with survival time after adjustment for age. Comparisons between groups at the time of TSH stimulation testing were made using the Kruskal‐Wallis test or using the Mann‐Whitney *U* test. Serum tT4 and TSH concentrations were compared pre and 12 months post LT4 supplementation using the Wilcoxon signed rank test. Systolic blood pressure was also compared over a 12‐month period in hypothyroid cats that were and were not supplemented with LT4 using the Wilcoxon signed rank test (excluding data from cats started on antihypertensive therapy). Data are presented as median [minimum‐maximum] unless otherwise stated, and statistical significance was defined as *P* < .05.

### 2.5 Ethical approval and statement of animal care

Exclusively nonexperimental (client‐owned) animals were used in this work. Ethical approval for the study was granted by the Douglas College Institutional Animal Care Committee (accredited by the Canadian Council on Animal Care). Informed consent was obtained in writing from the owner or legal custodian of all animals described in this work for the procedures undertaken.

## 3 RESULTS

From a study group of 949 RI treated animals, 117 cats were included in this study. Fifty‐four cats were female (46%), and 63 cats were male (54%); all were neutered and were 14 [8‐20] years of age.

Baseline TSH stimulation testing was performed 423 [86‐1286] days after RI treatment. Seventy‐one cats (61%) were categorized as euthyroid, 21 cats (18%) were classified as equivocal and 25 cats (21%) were classified as hypothyroid. Of the 117 included cats, 112 had a single dose of RI and 5 cats had 2 doses because of failure to attain euthyroidism after initial RI treatment. Four of the 5 cats that failed to attain euthyroidism had their 2nd dose of RI within 6 months and 1 cat approximately 12 months after the first RI treatment. Of the 5 cats that had 2 doses of RI, 3 were in the hypothyroid group and 2 were in the euthyroid group and all 5 had the baseline TSH stimulation test performed >6 months after the 2nd dose of RI. Baseline clinicopathological data stratified by thyroid and renal status are summarized in Table 1. At the time of TSH stimulation testing, 10 hypothyroid cats (40%) had noncritical illnesses including untreated systolic hypertension (n = 4), poorly controlled diabetes mellitus (n = 2), gastrointestinal disease (n = 1), hepatic disease (n = 1), grade 4 dental disease (n = 1), anemia (PCV 22%, n = 1), and a renal mass (n = 1). The medications received by hypothyroid cats were insulin (Lantus, Sanofi, Laval, Quebec; n = 2), ursodiol (n = 1), potassium supplementation (n = 2), and B12 supplementation (n = 1). Fourteen euthyroid and equivocal cats (15%) had noncritical illness at time of TSH stimulation testing including systolic hypertension (n = 5), grade 4 dental disease (n = 5), gastrointestinal disease (n = 2), hepatic disease (n = 2), and heart disease (n = 2). Medications received by euthyroid and equivocal cats included potassium supplementation (n = 5), gabapentin (n = 2), ursodiol (n = 2), maropitant citrate (Cerenia, Zoetis Canada, Kirkland, Quebec; n = 1), famotidine (n = 1), amlodipine (n = 4), benazepril (n = 2), lactulose (n = 1), amoxicillin (n = 1), cyclosporine (Atopica, Elanco Canada, Mississauga, Ontario; n = 1), buprenorphine (n = 1), phosphate binders (n = 2), ranitidine (n = 1), and atenolol (n = 1).

**TABLE 1:** Demographic, biochemical and blood pressure data for 117 cats at baseline after radioiodine (RI) therapy.

Variable	Euthyroid		Equivocal		Hypothyroid	
	Nonazotemic	Azotemic	Nonazotemic	Azotemic	Nonazotemic	Azotemic
Number	60	11	18	3	15	10
Time since RI (days)	413 [146‐1281]	675 [213‐1056]	267 [86818]	699 [163‐905]	423 [100‐1037]	532 [148‐992]
Age (years)	13 [8‐19]	15 [12‐20]*	14 [9‐19]	13 [13‐16]	15 [8‐19]	16 [12‐17]
Urea (mg/dL)	28.7 14.9‐42.7]	40.2 [32.0‐87.6]	29.1 [14.0‐83.4]	45.8 [43.0‐48.0]	29.8 [21.9‐46.6]	47.2 [33.4‐100.6]
Creatinine (mg/dL)	1.77 [0.85‐2.49]	2.71 [2.58‐3.92]	1.65 [0.42‐2.32]	2.77 [2.52‐3.46]	2.05 [1.30‐2.59]	3.12 [2.60‐5.17]
tT4 (μg/dL)	1.89 [0.71‐3.21]	1.66 [1.17‐2.01]	1.55 [1.13‐1.92]	1.59 [1.40‐1.80]	1.04 [0.27‐1.59]	1.04 [0.62‐1.22]
Free T4 (ng/dL)	1.14 [0.27‐2.41]	1.42 [0.78‐1.79]	0.86 [0.27‐1.50]	0.88 [0.62‐1.14]	0.37 [0.27‐1.50]	0.33 [0.27‐0.64]
TSH	0.14 [0.02‐5.67]	0.11 [0.02‐0.74]	0.28 [0.02‐4.58]	1.7 [0.39‐4.72]	0.99 [0.1‐12.0]	4.14 [0.13‐12.0]
USG	1.036 [1.013‐1.063]	1.016 [1.010‐1.026]	1.034 [1.013‐1.066]	1.015 [1.014‐1.022]	1.032 [1.014‐1.047]	1.017 [1.014‐1.025]
UPC	0.1 [0.1‐1.1]	0.1 [0.1‐1.3]	0.1 [0.1‐0.6]	0.2 [0.1‐0.3]	0.1 [0.1‐0.5]	0.15 [<0.1‐0.7]
SBP (mm Hg)	136 [95‐225]	126 [114‐150]	140 [95‐188]	135 [125‐165]	153 [92‐219]	144 [100‐206]

*Note*: Median [min‐max] values are given.

Abbreviations: BUN, blood urea nitrogen; RI, radioiodine; SBP, systolic blood pressure; TSH, thyroid stimulating hormone; TT4, total thyroxine concentration; USG, urine specific gravity.

* Denotes significant difference between groups (*P* = .005).

There was no difference in the time from RI treatment to TSH stimulation testing between hypothyroid, equivocal and euthyroid cats (443 [100‐1037] vs 274 [96‐905] vs 427 [146‐1281] days, respectively; *P* = .056, Table 1). Euthyroid azotemic cats were significantly older than euthyroid nonazotemic cats (Table 1; *P* = .005). There was no significant difference in age between hypothyroid azotemic and hypothyroid nonazotemic cats (*P* = .285), nor between equivocal azotemic and nonazotemic cats (*P* = .959). Follow‐up of cats was undertaken 12 months after TSH stimulation testing in 75 cats, none of which developed hyperthyroidism.

### 3.1 LT4 supplementation

Levothyroxine supplementation was administered to 13/25 hypothyroid cats (52%), of which 4 (31%) were azotemic and 9 (69%) were nonazotemic. The total daily dose of LT4 administered was 100 [50‐200] μg, delivered either q12h (n = 7) or q24h (n = 6). Levothyroxine supplemented cats were reevaluated 50 [38‐207] weeks after baseline sampling.

**FIGURE 1:**
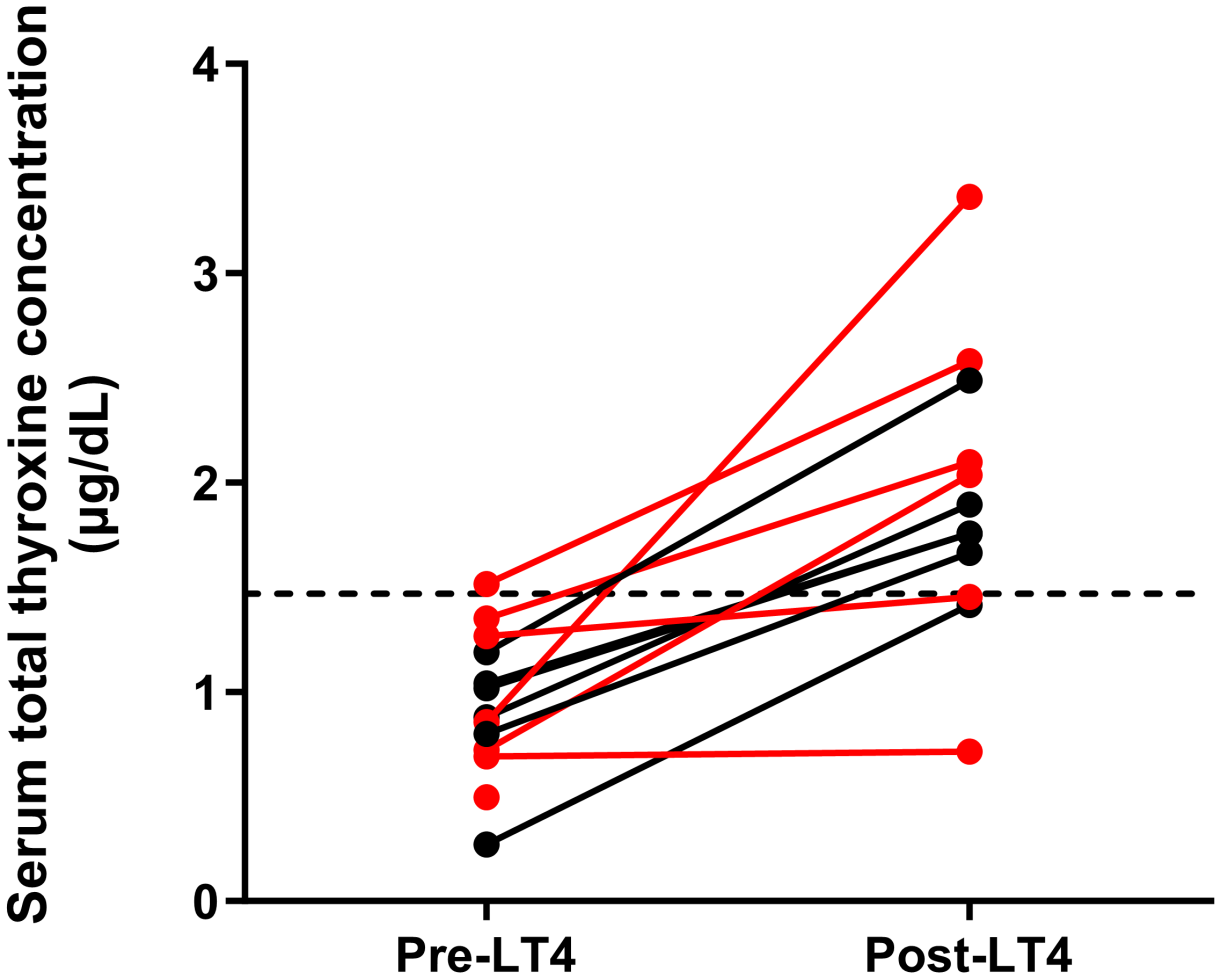
Line graph showing the change in serum total thyroxine (tT4) concentration (μg/dL) of levothyroxine (LT4) supplemented hypothyroid cats (azotemic and nonazotemic) pre and 12 months post LT4 supplementation. The dotted line represents the lower limit of the reference interval (1.5 μg/dL). Data points in black represent cats that were treated with once daily LT4, and data points in red represent cats that were treated with twice daily LT4. Total T4 concentrations increased significantly after supplementation (*P* = .003). LT4, levothyroxine.

At reevaluation (12 months post start of LT4 supplementation), the tT4 of supplemented hypothyroid cats had increased significantly (pretreatment 0.88 [0.27‐1.5] μg/dL, posttreatment 1.83 [0.71‐3.4] μg/dL, n = 11; *P* = .003, Figure 1), although tT4 remained below the reference interval (<1.5 μg/dL [19 nmol/L]) in 3 cats all of which were nonazotemic. One supplemented cat died before reevaluation and another cat with low tT4 posttreatment was reported to have run out of LT4 medication before follow‐up.

**FIGURE 2:**
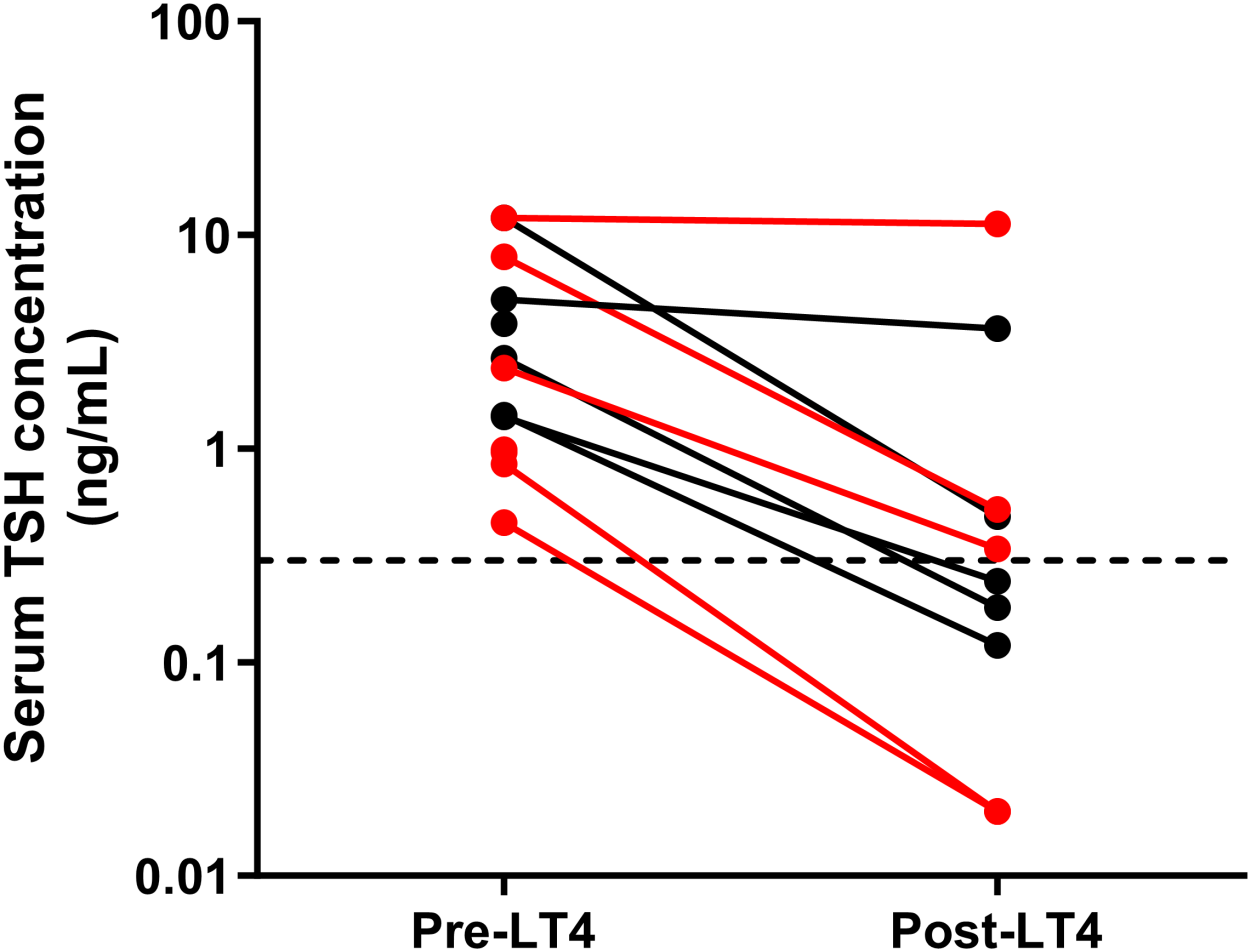
Line graph showing the change in serum thyroid stimulating hormone (TSH) concentration (ng/mL) of levothyroxine (LT4) supplemented hypothyroid cats (azotemic and nonazotemic) pre and 12 months post LT4 supplementation. The dotted line represents the upper limit of the reference interval (0.3 μg/dL). Data points in black represent cats that were treated with once daily LT4, and data points in red represent cats that were treated with twice daily LT4. Serum TSH concentrations decreased significantly after supplementation (*P* = .008). TSH, thyroid stimulating hormone; LT4, levothyroxine.

Serum TSH concentrations decreased significantly after 12 months of LT4 supplementation (2.65 [0.45‐12] ng/mL to 0.34 [<0.03‐11.3] ng/mL, n = 9; *P* = .008, Figure 2), although serum TSH concentration remained above the reference interval (>0.3 ng/mL) in 5 cats, 1 of which had a tT4 just below the reference interval (1.45 μg/dL). Four animals did not have serum TSH concentrations measured after LT4 treatment, including the 1 cat that died before reevaluation.

**FIGURE 3:**
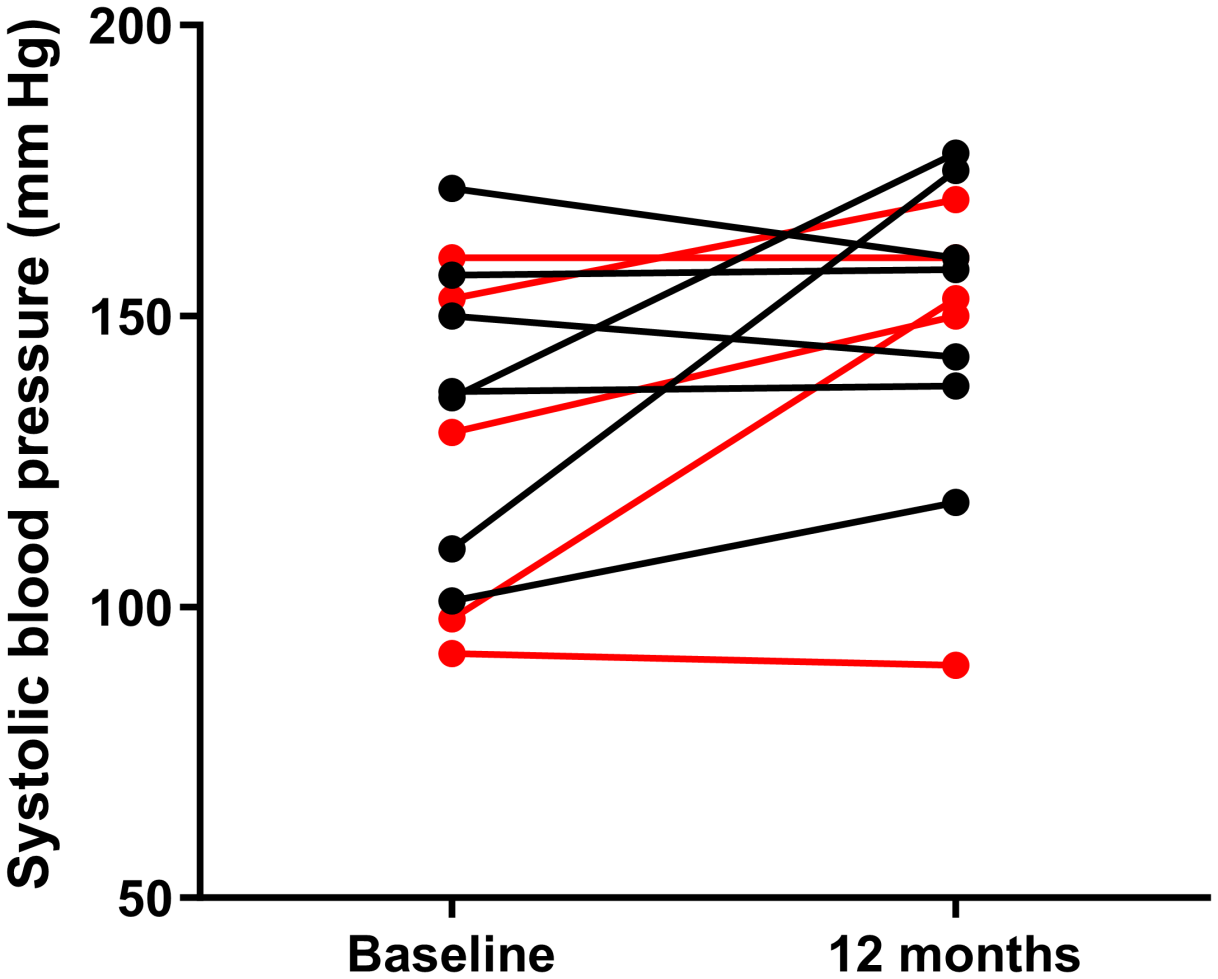
Line graph showing change in systolic blood pressure (SBP; mm Hg) between baseline and follow‐up testing in hypothyroid (azotemic and nonazotemic) cats that did (red) and did not (black) receive levothyroxine (LT4) supplementation during the follow‐up period. Systolic blood pressure did not change significantly in the nonsupplemented or supplemented group (*P* = .24 and *P* = .14, respectively).

Systolic blood pressure measurements were made 50 [38‐207] weeks after TSH stimulation testing in 8 supplemented hypothyroid cats (7 nonazotemic and 1 azotemic) and 7 nonsupplemented hypothyroid cats (5 nonazotemic and 2 azotemic). No hypothyroid cats had been treated with antihypertensive therapy before TSH stimulation testing. Three supplemented cats (38%, 2 nonazotemic and 1 azotemic), and zero nonsupplemented cats were started on antihypertensive therapy during the 12 month follow‐up period and were excluded from further analysis of SBP. Systolic blood pressure did not change significantly in the supplemented group (130 [92‐160] mm Hg vs 153 [90‐170] mm Hg, n = 5; *P* = .14; Figure 3), or nonsupplemented group (137 [101‐172] mm Hg vs 158 [118‐178] mm Hg, n = 7; *P* = .24; Figure 3) over the 12 month follow‐up period.

### 3.2 Survival analyses

Ninety‐five of 117 cats (81%) were euthanized or died before the study end point. Twenty‐two cats were alive at the end of the study (17 euthyroid, 3 equivocal, 2 hypothyroid; 19%).

**FIGURE 4:**
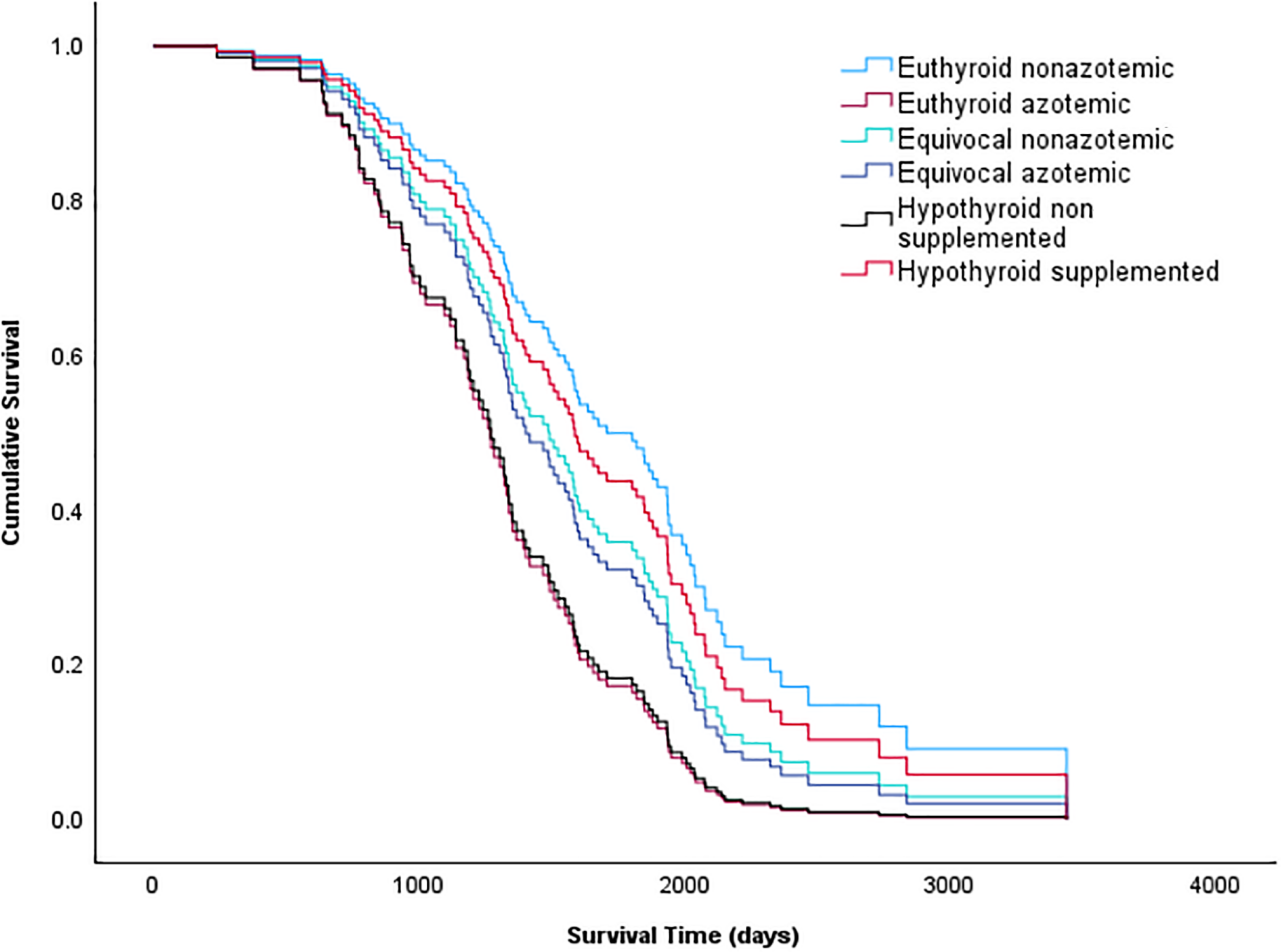
Survival curves of 117 cats from the time of radioiodine treatment separated into groups of: Euthyroid nonazotemic (blue), euthyroid nonazotemic (burgundy), equivocal nonazotemic (green), equivocal azotemic (purple), hypothyroid nonsupplemented (black), and hypothyroid supplemented (red) after adjustment for age. Euthyroid azotemic cats had significantly shorter survival times than the euthyroid nonazotemic cats after adjustment for age (HR 2.5, 95% confidence interval for HR 1.2‐5.2; *P* = .012). The hypothyroid nonsupplemented group also had significantly shorter total survival times than the euthyroid nonazotemic group after adjustment for age (HR 2.4, 95% confidence interval for HR 1.2‐4.8; *P* = .01).

Nonazotemic euthyroid cats had significantly longer survival times compared to azotemic euthyroid cats (1616 [663‐3369] days vs 934 [759‐2035]; *P* = .003) and also compared to nonsupplemented hypothyroid (combined azotemic and nonazotemic) cats (1232 [238‐2363] days; *P* = .002). The survival times in supplemented hypothyroid cats (azotemic and nonazotemic) were not increased compared to nonsupplemented hypothyroid (azotemic and nonazotemic) cats (1365 [525‐3438] days vs 1259 [238‐2363] days, respectively; *P* = .093). Multivariable Cox regression analysis showed that the euthyroid azotemic group had significantly shorter survival times than the euthyroid nonazotemic group after adjustment for age (HR 2.5, 95% confidence interval for HR 1.2‐5.2; *P* = .012, Table 2, Figure 4). The hypothyroid nonsupplemented group also had significantly shorter total survival times than the euthyroid nonazotemic group after adjustment for age (HR 2.4, 95% confidence interval for HR 1.2‐4.8; *P* = .01, Table 2, Figure 4).

**TABLE 2:** Multivariable Cox regression analysis of factors at time of TSH stimulation testing that were associated with survival time (time to death) from time of radioiodine therapy to death (all‐cause mortality).

Variable	*B*	Hazard ratio (HR)	95.0% CI for HR		Significance
			Lower	Upper	
Age	0.043	1.044	0.955	1.141	.342
Thyroid/renal status (referent—euthyroid nonazotemic, n = 60)					.067
Euthyroid azotemic (n = 11)	0.93	2.53	1.23	5.23	.012
Equivocal nonazotemic (n = 18)	0.39	1.48	0.81	2.68	.20
Equivocal azotemic (n = 3)	0.49	1.63	0.50	5.30	.42
Hypothyroid nonsupplemented (n = 12)	0.90	2.44	1.24	4.84	.010
Hypothyroid supplemented (n = 13)	0.18	1.19	0.59	2.40	.63

Abbreviations: SBP, systolic blood pressure; USG, urine specific gravity.

**FIGURE 5:**
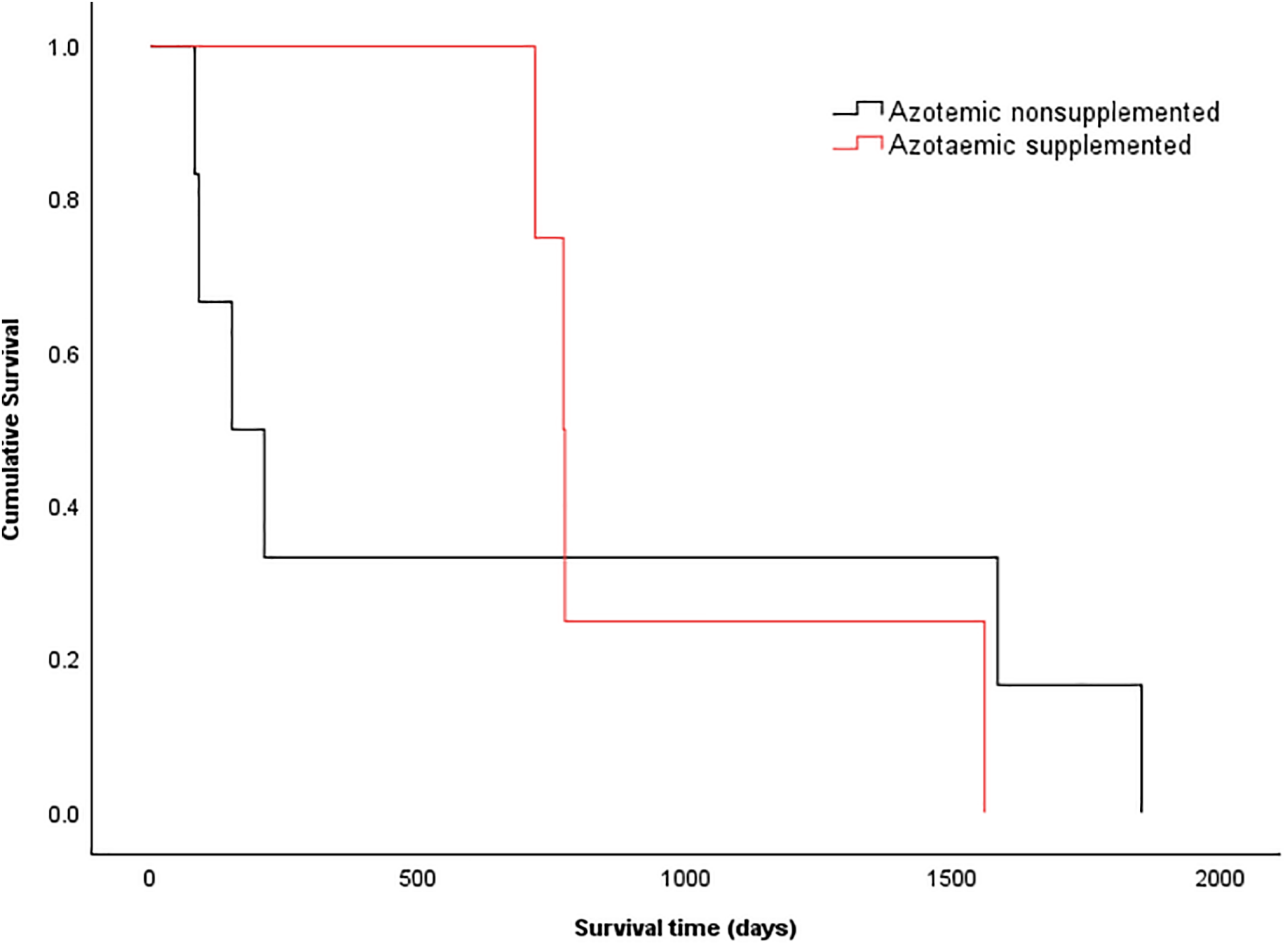
Kaplan‐Meier survival curve of cats from the time of TSH stimulation testing separated into groups of nonsupplemented hypothyroid azotemic (black, n = 6), and supplemented hypothyroid azotemic cats (red, n = 4). There was no difference in survival of supplemented and nonsupplemented hypothyroid azotemic cats (*P* = .991).

**FIGURE 6:**
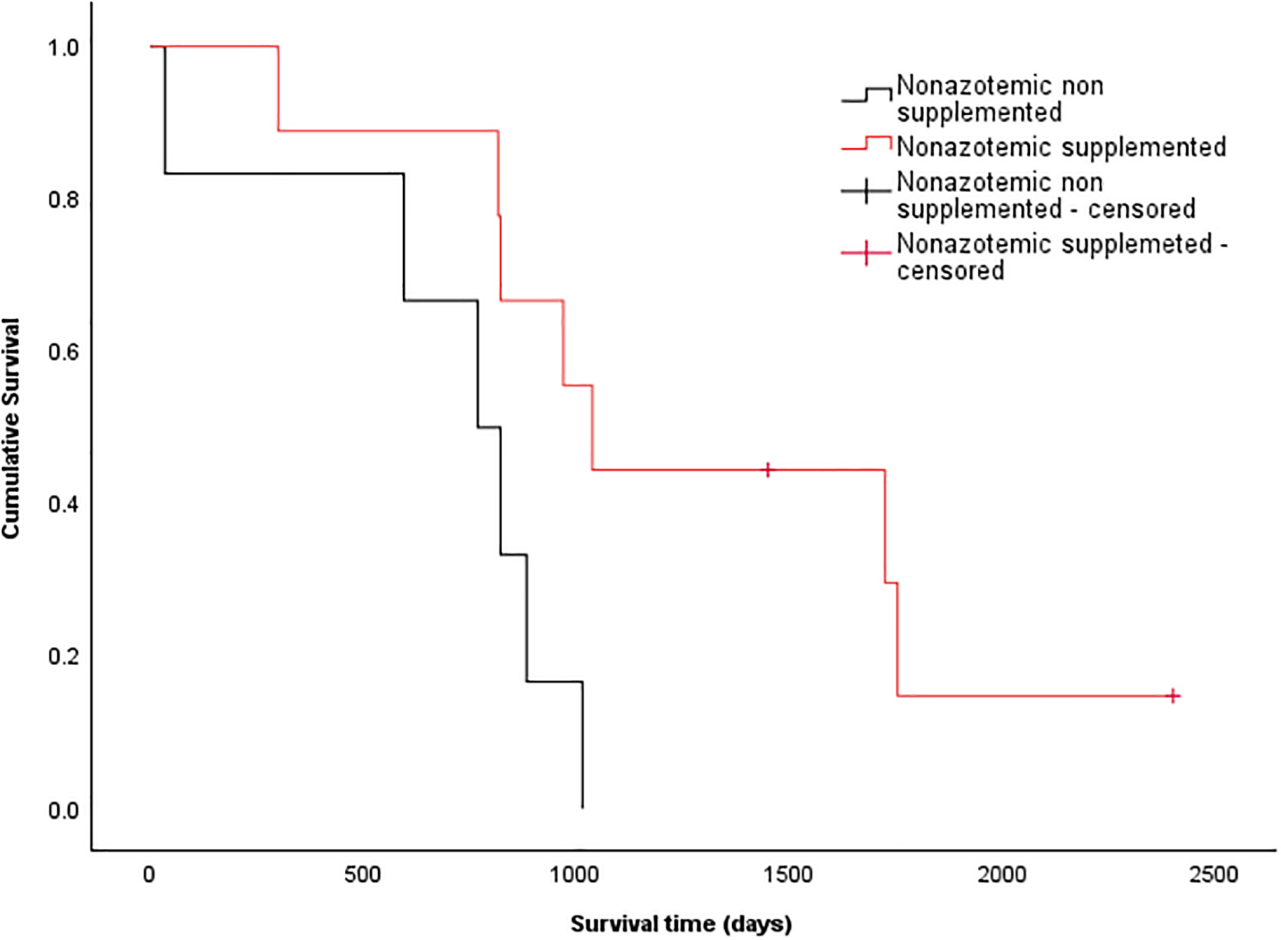
Kaplan‐Meier survival curve of cats from the time of TSH stimulation testing separated into groups of hypothyroid nonsupplemented nonazotemic (black, n = 6), and hypothyroid supplemented nonazotemic cats (red, n = 9). Hypothyroid supplemented nonazotemic cats had significantly longer survival times than hypothyroid nonsupplemented nonazotemic cats (*P* = .027).

When supplemented hypothyroid cats were stratified according to azotemic status at time of TSH stimulation testing, the nonazotemic supplemented cats were significantly younger than nonsupplemented nonazotemic cats (*P* = .027; Table 3). These cats also had significantly lower serum creatinine concentrations compared to the supplemented nonazotemic group (*P* = .021; Table 3). Serum urea concentrations were not significantly different between the supplemented and nonsupplemented nonazotemic groups (*P* = .16; Table 3). Age and serum concentrations of urea and creatinine were not significantly different between the supplemented and nonsupplemented azotemic cats (Table 3). Survival times of supplemented azotemic cats were not significantly longer than those of nonsupplemented azotemic cats (771 [718‐1558] days vs 152 [82‐1852] days, respectively; *P* = .991, Figure 5); however, survival times of supplemented nonazotemic hypothyroid cats were significantly longer compared with the nonsupplemented nonazotemic group (1037 [300‐2401] days vs 768 [34‐1014] days, respectively; *P* = .027, Figure 6).

**TABLE 3:** Baseline biochemical variables and systolic blood pressure of the hypothyroid group stratified by renal and LT4 supplementation status.

Variables	Supplemented nonazotemic	Nonsupplemented nonazotemic	Supplemented azotemic	Nonsupplemented azotemic
Number	9	6	4	6
Age	13 [8‐16]*	15 [14‐19]*	16 [14‐17]	15 [12‐17]
Creatinine (mg/dL)	2.09 [1.71‐2.48]^#^	1.56 [1.30‐2.30]^#^	3.14 [2.92‐4.75]	3.22 [2.60‐5.20]
Urea (mg/dL)	30.52 [26.0‐38.1]	54.3 [33.3‐100]	47 [33.6‐56.8]	26.3 [22.1‐46.5]
USG	1.031 [1.014‐1.047]	1.033 [1.014‐1.046]	1.015 [1.014‐1.017]	1.018 [1.014‐1.025]
SBP (mm Hg)	153 [92‐219]	146 [110‐172]	168 [127‐206]	139 [100‐170]

Abbreviations: SBP, systolic blood pressure; USG, urine specific gravity.

* Denotes significant differences between groups (*P* = .027).

^#^ Denotes significant differences between groups (*P* = .021).

## 4 DISCUSSION

The results of the present study indicate that posttreatment azotemia is associated with a reduced survival time in cats that are euthyroid after RI treatment. Furthermore, LT4 supplementation of nonazotemic hypothyroid cats (diagnosed using the TSH stimulation test)^10^ significantly increased survival time, although further studies using a larger group of age‐matched nonsupplemented hypothyroid nonazotemic cats would be required to confirm these findings.

Euthyroid nonazotemic cats in this study had median survival times of approximately 4.5 years, similar to the survival times for RI‐treated cats previously reported in the literature (2 to 4 years).^3^, ^4^, ^5^, ^6^, ^7^ However, cats that were azotemic and euthyroid after RI treatment had significantly reduced survival times compared to euthyroid nonazotemic cats, with the median survival time of euthyroid azotemic cats being around 2 years shorter than that of the euthyroid nonazotemic cats. These findings concur with those of a previous study of RI treated cats that also reported a negative association between posttreatment azotemia and survival.^6^ However, these results conflict with data that suggest that the development of azotemia in medically treated initially nonazotemic hyperthyroid cats that remain euthyroid post treatment is not associated with reduced survival times compared to cats that remain nonazotemic post treatment.^9^ This discrepancy might reflect the fact that in the aforementioned study hyperthyroid cats with azotemia before treatment were excluded, whereas in the present study, standardized assessment of renal function before treatment of hyperthyroidism was not possible because many cats that presented for RI treatment had been successfully managed using reversible hyperthyroid treatments before RI, whereas other cats presented without prior reversible treatment or after failed treatment attempts using medication or diet, and it is already well established that pretreatment azotemia reduces survival times.^5^, ^15^

Results of this study show that cats diagnosed as hypothyroid based on TSH stimulation test results,^10^ but that are not supplemented with LT4 (combining both azotemic and nonazotemic) had shorter survival times than nonazotemic euthyroid cats. The negative impact of hypothyroidism on survival could be theoretically attributed to the effects on erythropoiesis^20^ and cardiovascular function^21^ which are documented in the human literature, or, the effects of hypothyroidism on renal function.^8^, ^16^ However, interpretation of the association between hypothyroidism and survival times in the present study is complicated by the inclusion of both azotemic and nonazotemic cats in the nonsupplemented hypothyroid group, since hypothyroid cats with concurrent azotemia are expected to have reduced survival times compared to their hypothyroid nonazotemic counterparts.^9^ Further stratification of nonsupplemented hypothyroid cats by renal function was not performed in the present study because of the low numbers of cats in each group; however, further studies to compare the survival times of larger groups of hypothyroid nonsupplemented cats with and without azotemia with nonazotemic euthyroid cats would be of interest to provide further support to the negative association between hypothyroidism and reduced survival times.

This study found that LT4 supplementation in nonazotemic hypothyroid cats was associated with improved survival times compared to nonsupplemented nonazotemic hypothyroid cats (when hypothyroidism was diagnosed based on the TSH stimulation test).^10^ The effect of LT4 supplementation in RI treated nonazotemic hypothyroid cats has not previously been reported; however, a similar association was reported after restoration of euthyroidism in RI treated hypothyroid azotemic cats.^17^ Nonsupplemented cats in the present study were older than supplemented cats, which could confound our findings, although the nonsupplemented cats also had a lower serum creatinine concentration before LT4 supplementation, which could suggest better presupplementation renal function or could reflect a decreased muscle mass in these cats.^8^, ^22^ Moreover, LT4 supplementation was not randomized and the decision to treat could have been influenced by owner interpretation of preexisting quality of life, hence further randomized placebo‐controlled studies investigating LT4 supplementation in cases of iatrogenic hypothyroidism are warranted.

A significant survival benefit of LT4 supplementation in azotemic cats with iatrogenic hypothyroidism (diagnosed using the TSH stimulation test)^10^ was not demonstrated in this study which contrasts with the findings of a single previous study^17^; however, it is likely that our study was statistically underpowered to detect significant differences in survival time after LT4 supplementation in azotemic cats. Furthermore, normalization of serum concentrations of total T4 and TSH was not achieved in 7 of 13 hypothyroid cats after LT4 supplementation, which could suggest that euthyroidism was not achieved in many cases. However, increased serum TSH concentrations can occur in some euthyroid cats^10^, ^23^ and low tT4 concentrations can be associated with nonthyroidal illness such as CKD,^19^, ^24^ therefore, it is possible that euthyroidism was restored in these cats despite the lack of normalization in serum tT4 and TSH concentrations.

One previous study identified a higher SBP in cats with hypothyroidism compared to euthyroid cats,^10^ which could suggest that hypothyroidism is associated with hypertension in cats; however, a similar prevalence of hypertension was identified in hypothyroid and euthyroid cats after RI treatment in another study.^25^ Hypothyroidism in humans is associated with hypertension and, although this typically improves with supplementation of thyroid hormones, which implies a causal association, the mechanism for hypertension in hypothyroidism is not well understood.^26^, ^27^, ^28^, ^29^, ^30^ In the present study, SBP did not decrease after LT4 supplementation of hypothyroid cats (diagnosed using the TSH stimulation test),^10^ and differences in the proportion of cats started on antihypertensive therapy during follow‐up were also not evident between supplemented and nonsupplemented cats. Hence these results cannot provide support that low thyroid hormone concentrations are associated with increased SBP, although our study was likely underpowered to detect significant differences, and further studies with larger cohorts would be needed to confirm our findings.

In the present study we used the results of the TSH stimulation test rather than serum TSH and tT4 concentrations to classify cats as hypothyroid because some previous studies have demonstrated that elevated serum TSH concentrations (as determined using chemiluminescent assays) can be observed in cats diagnosed as euthyroid based on thyroid scintigraphy.^23^ The sensitivity and specificity of the TSH stimulation test for hypothyroidism (diagnosed using thyroid scintigraphy) is currently unknown and further investigation of the correlation between the 2 diagnostic tests would be warranted to support the use of the TSH stimulation test as a diagnostic test for iatrogenic hypothyroidism in cats. The administration of exogenous TSH should lead to a significant increase in tT4 production by thyrocytes in euthyroid cats, hence a lack of a significant increase in tT4 was interpreted as evidence of hypothyroidism in the present study. A false positive diagnosis of hypothyroidism would be possible if autonomous thyroid tissue, that was nonhyperfunctional but unresponsive to TSH, remained after RI treatment in some cats, and 1 preliminary study suggests that 23% of RI treated euthyroid cats have autonomous nonhyperfunctional thyroid tissue after RI treatment.^31^ Therefore, it is possible that some cats classified as hypothyroid based on TSH stimulation testing might have had sufficient residual nonhyperfunctional autonomous thyroid tissue to maintain biochemical euthyroidism; however, further studies to evaluate the prevalence of nonhyperfunctional but autonomous thyroid tissue after RI treatment are required to determine the possible impact of this on the diagnosis of iatrogenic hypothyroidism based on TSH stimulation testing.

In conclusion, the results of the present study demonstrate that azotemia is associated with shorter survival times in euthyroid cats, and LT4 supplementation after treatment might be beneficial in hypothyroid nonazotemic cats (when hypothyroidism is diagnosed using the TSH stimulation test).^10^

## CONFLICT OF INTEREST DECLARATION

Laboratory testing was performed at a reduced charge by IDEXX Laboratories Inc. IDEXX Laboratories Inc. staff were not involved in the study design, data collection and interpretation or the writing of the report. The authors declare no other conflicts of interest.

## OFF‐LABEL ANTIMICROBIAL DECLARATION

Authors declare no off‐label use of antimicrobials.

## INSTITUTIONAL ANIMAL CARE AND USE COMMITTEE (IACUC) OR OTHER APPROVAL DECLARATION

Granted by the Douglas College Animal Care Committee (accredited by the Canadian Council on Animal Care), and all procedures were performed after obtaining owner consent.

## HUMAN ETHICS APPROVAL DECLARATION

Authors declare human ethics approval was not needed for this study.
